# The Contributions of Onchocerciasis Control and Elimination Programs toward the Achievement of the Millennium Development Goals

**DOI:** 10.1371/journal.pntd.0003703

**Published:** 2015-05-21

**Authors:** Caitlin Dunn, Kelly Callahan, Moses Katabarwa, Frank Richards, Donald Hopkins, P. Craig Withers, Lucas E. Buyon, Deborah McFarland

**Affiliations:** 1 Health Programs, The Carter Center, Atlanta, Georgia, United States of America; 2 Hubert Department of Global Health, Rollins School of Public Health, Emory University, Atlanta, Georgia, United States of America; 3 College of Arts and Sciences, Emory University, Atlanta, Georgia, United States of America; University of Queensland, AUSTRALIA

## Abstract

In 2000, 189 member states of the United Nations (UN) developed a plan for peace and development, which resulted in eight actionable goals known as the Millennium Development Goals (MDGs). Since their inception, the MDGs have been considered the international standard for measuring development progress and have provided a blueprint for global health policy and programming. However, emphasis upon the achievement of priority benchmarks around the “big three” diseases—namely HIV, tuberculosis (TB), and malaria—has influenced global health entities to disproportionately allocate resources. Meanwhile, several tropical diseases that almost exclusively impact the poorest of the poor continue to be neglected, despite the existence of cost-effective and feasible methods of control or elimination. One such Neglected Tropical Disease (NTD), onchocerciasis, more commonly known as river blindness, is a debilitating and stigmatizing disease primarily affecting individuals living in remote and impoverished areas. Onchocerciasis control is considered to be one of the most successful and cost-effective public health campaigns ever launched. In addition to improving the health and well-being of millions of individuals, these programs also lead to improvements in education, agricultural production, and economic development in affected communities. Perhaps most pertinent to the global health community, though, is the demonstrated effectiveness of facilitating community engagement by allowing communities considerable ownership with regard to drug delivery. This paper reviews the contributions that such concentrated efforts to control and eliminate onchocerciasis make to achieving select MDGs. The authors hope to draw the attention of public policymakers and global health funders to the importance of the struggle against onchocerciasis as a model for community-directed interventions to advance health and development, and to advocate for NTDs inclusion in the post 2015 agenda.

## Introduction

As the 2015 deadline to achieve the Millennium Development Goals (MDGs) nears, it is timely to assess achievements and consider new priorities now being set for the post-2015 agenda. Although data limitations have led to some concerns over their utility in gauging the equity and sustainability of achievements [1–4], the MDGs are considered to be the international standard for measuring development progress. Moreover, since their inception, the MDGs have guided global health policy and programming, as they illustrate the link between population health and development more broadly [4,5]. This association is nowhere more evident than in the impact of Neglected Tropical Diseases (NTDs) on the world’s poorest populations. As both proxies for and promoters of poverty, NTDs act as an impediment to human development; hence, a concerted effort to control and eliminate these diseases would make an indelible mark on the health and well being of the very poor [6].

Of the major NTDs, onchocerciasis is one of the most common and detrimental. It is endemic to 31 African countries as well as 6 countries in the Americas and several areas in Yemen, putting over 100 million people at high risk of onchocerciasis infection worldwide [7]. Of the estimated 37 million people currently infected, 99% live in sub-Saharan Africa [8,9]. Onchocerciasis is transmitted by biting black flies, of the genus *Simulium*, found near the fast flowing waters in which they breed—hence its common name, river blindness. When taking a blood meal, the black fly deposits infective larvae of a nematode worm, *Onchocerca volvulus*, into its human host [10]. Upon maturation, female worms release thousands of embryos, called microfilariae (mf), per day, for the duration of their nine to 11 year reproductive lifespan [8,10,11]. When the mf die, they invoke an inflammatory immune response; this repeated reaction destroys tissue and causes damage to the eyes, skin, and, possibly, the brain [12].

Onchocerciasis is the second leading infectious cause of preventable blindness [13]. It causes an array of serious morbidities, including intense itching, Onchocercal Skin Disease (OSD)[14], musculoskeletal pain and general malaise [15], weight loss, and “hanging groin,” or elephantiasis of the genitals [16,17]. It is further suspected to be a cause of epilepsy [12,18]. Beyond health, onchocerciasis leads to grave social and economic consequences that exacerbate poverty and hinder overall development [13,18,19].

Efforts to control onchocerciasis through weekly aerial spraying of larvicides began in West Africa in the early 1970s under the management of the Onchocerciasis Control Program (OCP). This initiative was extremely successful, eliminating onchocerciasis as a disease of public health importance from 10 of the 11 OCP countries [8,20]. With the discovery of a safe and effective microfilaricide, ivermectin (brand name Mectizan), and its subsequent donation in 1987 by the pharmaceutical company Merck & Co., onchocerciasis control was expanded to endemic countries in Central, East, and West Africa under the management of the African Program for Onchocerciasis Control (APOC) as well as the six endemic countries in Latin America through the Onchocerciasis Elimination Program for the Americas (OEPA) [20,21]. The disease is now controlled primarily through Mass Drug Administration (MDA) of ivermectin; one oral dose annually kills 95% of the mf, relieving most of the symptoms and temporarily reducing reproduction, thus slowing transmission [8]. However, with this treatment regimen, it is unclear when treatment can be halted without recrudescence. Elimination programs in some countries are beginning to implement twice yearly treatment with ivermectin as it has been shown to interrupt transmission in 6.5 years [22].

## Methods

To determine the impact onchocerciasis control and elimination programs have on the MDGs, the authors conducted a literature search using Pubmed, Web of Science, and Cab Direct. The terms “onchocerciasis AND river blindness” were searched in combination with the following key words: “MDGs,” “poverty,” “economics,” “malnutrition,” “food insecurity,” “education,” “helminth infections,” “children,” “school attendance,” “women,” “gender,” “gender equity,” “stigma,” “maternal health,” “reproductive health,” “mortality,” “loa loa,” “environment,” “HIV,” “partnerships,” “ivermectin,” “Mectizan Donation Program,” and “community directed.” Abstracts and full articles were reviewed if they addressed any of the MDG goals or targets. The reference sections of key sources were used to identify primary studies and other relevant articles. Additionally, publicly available data from APOC and the Mectizan Donation Program and reports from organizations that manage onchocerciasis programs were reviewed to assess the collective reach of current onchocerciasis partners.

These findings were used to populate a table divided by MDG targets and indicators (See Table 1). An MDG was selected for inclusion if any of its indicators appeared to be hindered by the presence of onchocerciasis or impacted by its control. Using this criterion, Goal 4 (reduce child mortality), Goal 5 (improve maternal health), and Goal 7 (ensure environmental sustainability) were excluded, as we found no evidence of an association with any indicators. Analyses from the United Nations (UN) Statistics Division, Center for Global Development, Overseas Development Institute, and African Development Bank were used to assess MDG progress among onchocerciasis endemic countries. However, since the MDGs are national indicators and onchocerciasis programs are restricted to relatively limited endemic foci, it was not feasible to parse out or quantify the degree that benefits achieved at the local level accrue to a national level. While not quantifiable, this article presents observations based on impact studies, economic evaluations, and qualitative reviews.

**Table 1:** The Impact of Onchocerciasis Control and Elimination Programs on the MDGs

MDG	Impact of Onchocerciasis	Impact of Onchocerciasis Programs and Disease Reduction
MDG 1: Eradicate extreme poverty and hunger	Agricultural production losses due to migration from fertile lands [8,18]	Vector control programs allow 25 million hectares to be reclaimed, enough food to feed over 18 million [8,13]
	Decreased worker productivity due to severe visual impairment and associated pain and fatigue [13,19]	Treatment with ivermectin reduces morbidities; [8] continuation of productive work [23]; OCP found to have Economic Rate of Return (ERR) of 20% per year; APOC ERR of 17% a year [8,19]
	Affected individuals spent more time and money seeking healthcare; medical poverty trap [18,19,24]	Treatment with ivermectin reduces morbidities; [8] time and money can be redirected
MDG 2: Achieve universal primary education	Children removed from school to care for affected relatives [19]	Reduced prevalence of onchocerciasis in the community; students stay in school [23]
	Severe itching and fatigue affect ability to concentrate and learn [18]	Ivermectin relieves itching from onchocerciasis and reduces ectoparasitic skin infections and certain intestinal parasites; student’s ability to focus and learn improved [5,13,25]
MDG 3: Promote gender equality and empower women	Stigma associated with disease prevents women from being married; impacts mental health [26–28]	Reduced morbidities allow women to participate freely in community life [29]
	Girls more likely to be removed from school to care for affected relatives [19]	Reduced prevalence of onchocerciasis in the community; girls stay in school [19]
		Recruitment of Community-Directed Distributors (CDDs); women empowered to effect change within the community
MDG 4: Reduce child mortality	(No known impact)	Reduced morbidities allow women to provide prolonged breastfeeding protection to children [30]
		Reduced prevalence of onchocerciasis in the community; children under the age of five less likely to become infected, improving development outcomes (helminth infection associated with impaired immune response) [31–33]
MDG 5: Reduce maternal mortality	(No known impact)	CDDs provide health education, including family planning [34]
MDG 6: Combat HIV/AIDS, malaria, and other diseases	Onchocerciasis as a disease; second leading infectious cause of blindness in the world; morbidities include OSD, hanging groin, weight loss, musculoskeletal pain, insomnia and fatigue [13,18,19]	Community-Directed Treatment with Ivermectin (CDTI) and vector control strategies; reduced prevalence of onchocerciasis in the community
	Helminth infections impairs immune response and ability to serocovert after vaccination; co-infection with NTDs and “big three” may worsen outcomes for patients [5,9,13,32,35]	Ivermectin reduces helminth burden [36,37]
		CDTI system facilitates integration with other health programs; improved coverage and uptake of services; cost savings [34,35,38–41]
		Strengthens health systems through operational research, training of CDDs and health workers and expanded distribution lines [5,21,25,34,42,43]
		Treatment with ivermectin confers secondary benefits; effective against certain intestinal parasites and ectoparasitic skin infections [5,13,25]
MDG 7: Ensuring environmental sustainability	(No known impact)	Vector control areas make use of environmentally safe larvicides; MDG is not impeded
MDG 8: Develop a global partnership for development	(No known impact)	Increased access to the drug, ivermectin, through drug donation by Merck [24,43]
		MDP is one of the largest public-private partnerships ever created; first of its kind and paved the way for similar programs. [25,34]

## Results

A literature and data review found that reducing the burden of onchocerciasis in highly endemic areas through control and elimination programs contributes to the achievement of several MDGs. This impact is explained in detail below and summarized in Table 1.

### MDG 1: Eradicate Extreme Poverty and Hunger

The initial epidemiological research into the extent of onchocerciasis in West Africa found the socioeconomic consequences of the disease to be devastating. Over 60% of the savannah population was infected, half of the men over the age of 40 were blind or visually impaired, and the disease was already affecting children [8]. In an attempt to escape this scourge, entire villages moved away from the fertile river valleys that were home to the black fly. Living in over-crowded villages with poor soil and little access to water, agriculture yields were reduced, and, subsequently, families were pushed further into poverty [8].

Research demonstrates that onchocerciasis exacerbates poverty in three main ways: by decreasing worker productivity and earnings, reducing agricultural yields, and increasing medical expenses [19,24]. A study on the economic impact of OSD at the second largest coffee plantation in Ethiopia found that employees with severe OSD earned 15% less in daily wages and missed, on average, an additional 1.9 days of work per month [19,24]. The difference in income represented 5.2% of the per-capita GDP at the time [24]. Exhaustion from sleep deprivation and musculoskeletal pain were found to impact productivity and absenteeism [13,18].

Individuals with onchocerciasis also spend more time and money seeking healthcare [13,19]. A multi-country study in Nigeria, Ethiopia, and Sudan found that those with severe OSD devote, on average, an additional $20 per year to health expenditures—up to 15% of their annual income. They were also found to spend more time in activities classified under “sickness” and “fatigue/weakness,” and less time in “productive” and “household” activities. On average, they spent 6.75 extra hours seeking health care over six months compared to their non-affected counterparts [18,19,24]. These added costs can push people into a downward economic spiral (the so called “medical poverty trap”), further widening inequalities [44].

There have been numerous economic evaluations of onchocerciasis control programs, all of which conclude that they have a high return on investment [13,24]. One of the most comprehensive analyses, conducted by the World Bank, calculated an economic rate of return (ERR) of 20% per year for the duration of the OCP, with 25% attributed to increased labor force and 75% to the increased land availability [8]. A 10% ERR is considered to be the standard of a successful development program [8,24]. The evaluation found that controlling onchocerciasis in West Africa allowed communities to reclaim 25 million hectares, enough land to feed 18 million people [13].

The World Bank estimated the ERR for APOC to be 17% a year. However, this was calculated using only the projected benefits from reductions in blindness and increases in land availability. It did not take into account benefits from reductions in other morbidities, such as OSD, which represents a larger portion of the Disability-Adjusted Life Year (DALY) burden in the region [8,19]. Even so, it was estimated that every US Dollar (USD) invested in the program between 1996 and 2017 would add 27 productive workdays [8]. The findings of these economic evaluations are supported by the experiences of affected communities. For instance, 75.6% of the respondents in Cameroon, the Democratic Republic of the Congo (DRC), Nigeria, and Uganda listed work productivity and improved food security as benefits of taking ivermectin [23].

These studies compellingly illustrate not only that onchocerciasis can affect a region’s economic development by debilitating the workforce and reducing land utilization but also that control and elimination programs can reverse the situation.

### MDG 2: Achieve Universal Primary Education

Onchocerciasis begins to take its toll in early childhood. In the early 1990s, in the Benue State of Nigeria, 30% of children aged five years and living in hyperendemic regions had some level of visual impairment due to onchocercal eye disease [18]. Another study, conducted in the Taraba River Valley of Nigeria, found that children as young as five had skin lesions and some students had irreversible eye lesions affecting their vision [27]. Academic performance was found to be negatively correlated with infection as lost vision, constant itching, insomnia, and fatigue affected students’ ability to work [18].

A World Bank study found the risk of dropping out of school was twice as high for children whose head of household had OSD and girls were more likely to be taken out of school to care for a disabled relative than boys [19]. Following treatment with ivermectin, nearly a third (29%) of those surveyed in Cameroon, the DRC, Nigeria, and Uganda mentioned that school attendance improved, as fewer children left school to care for sick relatives. Interviews and focus groups with school managers and parents revealed that children were able to focus more as their skin cleared and itching was alleviated [23].

A major and largely unmeasured benefit of MDA with ivermectin is that the drug is also effective against scabies, lice, bed bugs, and certain intestinal parasites [5,25,45,46]. Studies have found that treating children with anti-helminthic medications increases appetite, weight, and height, and leads to increases in school attendance [13]. Though it is difficult to determine the attributable effect of ivermectin in areas that are also treated for Soil-Transmitted Helminths (STH), it is clear that students feel relief after treatment.

### MDG 3: Promote Gender Equality and Empower Women

A disproportionate burden of the disease falls on women and girls in relation to caretaking and stigma [26–28]. In many societies, marriage is considered an important cultural rite of passage and can be central to female identity. By causing skin deformities, OSD damages marriage prospects, and is, as a result, perceived to affect a woman’s future well-being [26,29]. In fact, in parts of Nigeria, onchodermatitis is known as “osepuru nwanyi aka na di” or the disease that “prevents a girl from getting married” [47]. These concerns are validated by studies that found women with the highest levels of onchocercal infection were more likely to be single despite being of marriageable age [18], and that girls with skin lesions were avoided and married later in life [30].

Cultural beliefs about disease etiology also contribute to the ostracism of infected women. In parts of West Africa, it is believed that the afflictions of a mother will be passed to her children [18,47]. Studies in both South America and Africa found many people believe onchocerciasis causes reproductive complications including spontaneous abortion, stillbirth, and infertility [27,48]. While not substantiated with evidence, these beliefs still have bearing since a woman’s perceived reproductive abilities are considered important in assessing suitability for marriage [49].

In addition to facing these negative cultural responses, women with onchocerciasis also appear to be affected by unique pathologies. For example, some women develop a “hanging groin.” Women with such visible symptoms have low self-esteem, experience embarrassment, and avoid community activities [27]. Though stigma affects both genders, girls are more likely to withdraw from social activities, including school, to avoid the shame associated with lesions [29].

### MDG 6: Combat HIV/AIDS, Malaria, and Other Diseases

Not surprisingly, the onchocerciasis programs most directly address MDG 6. A host of reports highlight the successes of OCP, APOC, and OEPA in alleviating the worst manifestations of the disease in millions. However, the success of these programs extends beyond disease specific endeavors. These programs have also contributed to the development of rapid disease assessment and mapping techniques, the advancement of community-directed delivery systems, and an approach to public—private partnerships involving the pharmaceutical industry. The global onchocerciasis control and elimination effort has offered ancillary benefits of strengthened health systems and new models for other health and development programs [25,42,43].

The Community-Directed Treatment with Ivermectin (CDTI) system provides many opportunities for integration with other health programs. Already, Community-Directed Distributors (CDDs) have been involved in health activities, such as the Expanded Program on Immunization (EPI), malaria bed-net distribution and at-home treatment, Vitamin A distribution, and family planning counseling. In 2012 alone, more than 47 million treatments and commodities were delivered through the CDTI network, supporting 13 additional health interventions [34].

Integrating ivermectin distribution with other health interventions is not only possible but it also increases uptake and coverage, decreases costs, and makes programs more sustainable [38–41,50]. A pilot study in Nigeria found that integrating delivery of Vitamin A with ivermectin distribution increased coverage rates of Vitamin A substantially—from zero to 30% to an average of 80% [51]. WHO conducted a three-year multi-country study that also found integrated delivery with ivermectin is more effective than the regular delivery methods for malaria treatment, Insecticide Treated mosquito Nets (ITNs), and Vitamin A distribution. The additions to CDTI not only enhanced coverage for the add-ons but the onchocerciasis programs also saw increases of 10% [40]. Similarly, a study from The Carter Center and the United States Centers for Disease Control and Prevention found a nine-fold increase in ownership of Insecticide-Treated Mosquito Nets (ITNs) among households with children under the age of five and pregnant women when distribution was combined with CDTI, and there were no adverse effects on the onchocerciasis program goal [50]. The kinship-enhanced community-directed model, piloted in Uganda, which has CDDs distribute ivermectin to their relatives, has shown even better results in terms of coverage [52]. Furthermore, when drug delivery for NTD control is integrated, studies have found significant cost-savings [35,41].

The CDTI strategy has strengthened health systems by creating and strengthening distribution lines. In particular, it provides a viable entry point into remote and conflict-affected areas [5]. For example, in the Central African Republic, community distributors are some of the only health workers who reach all villages in certain areas [25]. CDDs have also played a valuable role in operational research and national surveillance [53]. For example, CDDs have gathered data of value for other programs and studies, such as the latitude and longitude of each village and health center, and the demographic characteristics of households in 32,000 communities [21]. In 2012, over 650,000 CDDs were trained or re-trained in 22 countries, as were over 80,000 health workers in 20 countries [34]. The increase in human capacity resulting from the mobilization and training of so many community-level volunteers is an un-measured, yet likely significant, contribution. The understanding of public health and sense of empowerment gained through their work will enable these community members to respond to other public health challenges.

In addition to the benefits of the CDTI system, onchocerciasis control and elimination may contribute to the overall health of affected populations. In sub-Saharan Africa, the same populations affected by HIV, tuberculosis (TB), and malaria are often polyparasited with NTDs [13]. Research now shows that NTDs render individuals more susceptible to the “big three,” and that coinfection may worsen outcomes for patients [5,13,35]. Helminth infections are thought to weaken the immune response and may impair the ability to seroconvert after vaccination, leaving infected individuals more vulnerable to diseases [9,32]. In children, intense infection may cause systemic effects such as epilepsy, growth retardation, and general debilitation [9,33]. MDA with ivermectin for onchocerciasis also confers secondary benefits on the targeted populations [37,45]. While albendazole is WHO’s recommended drug for STH, ivermectin is effective against ascaris and trichuris as well, and is the drug of choice for treating human strongyloidiasis [5,25,45]. MDA has also shown a reduction in prevalence of ectoparasitic skin infections, such as lice, bed bugs, fleas, and scabies, and the secondary skin infections they cause [5,46]. Scabies, a common mite infection seen in impoverished and overcrowded settings, is now considered an important risk factor for staphylococcus aureus and group A streptococci (GAS), the latter of which is associated with post-streptococcal glomerulonephritis (PSGN), a potential cause of renal disease, and possibly rheumatic fever [46]. Topical treatments prescribed often have low compliance and are particularly poorly tolerated in infants; oral ivermectin, however, appears to be an effective and safe alternative to treat recalcitrant scabies [54]. Moreover, ivermectin is a popular drug as it provides rapid relief from itching associated with onchocerciasis and other parasites [25]. Affected individuals attest that the drug improves overall social, psychological, and economic well-being [23].

### MDG 8: Develop a Global Partnership for Development

As a collaboration between WHO, the UN, the World Bank, the Food and Agriculture Organization, Pan American Health Organization, Merck, 31 African countries, 6 Latin American countries, 21 bilateral and multilateral donors, over 20 international and local Non-Governmental Organizations (NGOs), and over 190,000 endemic communities, the collective effort to eliminate onchocerciasis is an exemplary illustration of the global partnerships envisioned in MDG 8 [25,34].

Specifically, one of the targets under MDG 8 states that “in cooperation with pharmaceutical companies, provide access to affordable essential drugs in developing countries” [55]. In 1987, Merck began donating ivermectin through the Mectizan Donation Program (MDP) for “as long as needed” to help fight river blindness [20]. Merck later extended this donation to include treatment for lymphatic filariasis (LF), an NTD that requires treatment with both ivermectin and albendazole (donated by GlaxoSmithKline) [25]. Merck bears the cost of production, transportation to the port of entry, and any clearing costs, while the MDP manages administration and distribution of the drugs to the entities responsible for implementation [24]. Merck has calculated the value of each tablet to be $1.50, which would put their donation over the past twenty years at over two billion dollars [24,43].

Merck’s long-term commitment is one of the key reasons for the existence and success of the onchocerciasis programs. It allowed governments and NGOs the time to invest in establishing and sustaining efficient ivermectin-based MDA programs without the threat of the loss of ivermectin [25]. The MDP was the first of its kind and paved the way for similar NTD programs based on MDA, often with other donated drugs. It is the longest running drug donation program, and the onchocerciasis initiative continues to be one of the largest public—private partnerships ever created [25].

## Discussion

The MDGs aim to reduce extreme poverty and ensure equity in fundamental rights [55]. As demonstrated in this paper, onchocerciasis programs support these exact aims. By reducing the prevalence of a stigmatizing and disabling disease, onchocerciasis programs improve the overall health of individuals. In turn, this allows for gains in worker productivity, gender equity, and education, thus stimulating the development of affected communities.

Despite the crosscutting impact of onchocerciasis and other NTDs on human development, NTD programs have been largely underfunded and undervalued by the global health community. Instead, the primary focus of the past decade has been on the three diseases listed in MDG 6 [5,6,56]. A glance at the allocation of funds by global health entities illustrates this disparity: while HIV/AIDS programs received a full 37% of total international development assistance in 2010, NTDs were allocated a mere 0.6% [57]. While, in recent years, there has been greater recognition of the poverty-promoting attributes of NTDs, as well as the possibilities for cost savings through integrated NTD control (most notably through the resolutions of the Sixty-Sixth World Health Assembly and the London Declaration on NTDs), this has come from a limited group and has not resulted in the same call to action from the wider development community as with the response to the MDGs [28,58].

Looking towards the post-2015 agenda, it is imperative that the global community evaluate past development efforts, and build upon and invest in those that work. Modest investment in reaching “low hanging fruits” by delivering proven and cost-effective strategies makes sense and will pay back large dividends. Since these poverty-promoting diseases affect nearly one billion people [59], and are thought to worsen the health outcomes of those coinfected with priority diseases, the benefits of NTD control would likely extend beyond disease-specific targets. Furthermore, many NTD programs offer opportunities for integrated health delivery. For instance, the addition of multiple health interventions to community-directed treatment of ivermectin led to increased coverage and decreased operational costs. However, since onchocerciasis is only present in limited geographic zones, the cost and feasibility of scaling-up CDTI to the national level is unknown. In spite of this, more studies and reviews to document the contributions of onchocerciasis control and elimination programs are required in order to understand their contribution toward MDGs.

Onchocerciasis control and elimination efforts are widely considered among the most sustained, successful and cost-effective public health campaigns ever launched [9,20]. Their 40-year history provides valuable lessons, including: the need for rapid disease mapping to prioritize treatment areas, rigorous monitoring of impact, ensuring adequate coverage, establishing strong multilateral partnerships, and allowing freedom for each partner to focus on their specialty while benefiting from the experiences and expertise of a team. Perhaps most pertinent to the global health community, though, is the demonstrated effectiveness of facilitating community engagement, by allowing communities considerable ownership of drug delivery. With a network of over 650,000 CDDs throughout 190,000 communities [7], onchocerciasis control and elimination programs have strengthened national health care delivery systems across Africa, while empowering individuals to take an active role in improving their health, and the health of their families, neighbors, and communities.

While onchocerciasis control in much of Africa is indisputably a major public health achievement, evidence of interrupted transmission in many endemic regions in the Americas and several foci in Africa raises hopes for eventual elimination of the disease, which would be the ultimate sustainable solution for river blindness [60]. Moving towards a goal of elimination, it will be necessary to address the technical challenges of MDA in areas co-endemic with loiasis. Ivermectin is contraindicated for individuals coinfected with loiasis as it can cause severe adverse reactions, including encephalopathy. While this could potentially limit the expansion of MDA in parts of Cameroon, the Central African Republic, the Congo, the DRC, Nigeria, and South Sudan, which are endemic to this filarial disease, operational research suggests that comprehensive disease mapping and “pre-treatment” with albendazole could substantially reduce the risks associated with treatment [9,10,61]. Reaching adequate treatment coverage in regions with ongoing conflicts will also remain a major challenge to an African elimination agenda [12].

More research is urgently needed to develop a safe and effective macrofilaricide to kill adult worms, as well as develop better diagnostic tests [8–10,12]. In the meantime, mathematical models suggest that moving to semi-annual treatment, and vector control where feasible, will drastically reduce the time frame required to interrupt transmission [22,62]. Though scaling-up treatment is a daunting task given the expansive, often difficult to reach treatment areas and the existing constraints on health systems, several studies have shown that increasing treatment frequency has actually led to better coverage rates [63–66]. Rather, the sustainability of MDA is most threatened by the success of the CDTI platform; as more programs rely on community volunteers, they risk over-burdening CDDs and causing attrition. As APOC moves to increase the number of distributions per year, it is prudent to reconsider providing incentives to offset the hardship placed on volunteers. In sum: though highly efficient vectors and the vast terrain of affected regions in Africa represent considerable challenges, with adequate resources and the persistence of all involved, movement towards elimination is possible. Sufficient political will and stakeholder commitment to these efforts can make it so that future generations will never again suffer the fate of river blindness.

**Box 1: Key Learning Points**

- Neglected Tropical Diseases (NTDs) impact approximately 2.7 billion people worldwide, the majority of whom live in low-income, developing nations.
- Onchocerciasis, commonly known as river blindness, is a debilitating and stigmatizing NTD that causes a host of serious morbidities including blindness and Onchocercal Skin Disease (OSD).
- Programs to control onchocerciasis contribute to the United Nations Millennium Development Goals. In addition to improving the health and well-being of millions of individuals, these programs also lead to improvements in education, agricultural production, and economic development in affected communities.
- Onchocerciasis control and elimination programs have a well-documented history of success that offers invaluable lessons and best practice in public health; including, focus on data-driven programming, strong global partnerships, and a movement towards community ownership through the system of community-directed treatment.

**Box 2: Key Papers in the Field**

- Norris J, Adelman C, Spantchak Y, Marano K (2012) Social and Economic Impact Review on Neglected Tropical Diseases Washington, DC: The Hudson Institute. 36 p.
- Benton B (1998) Economic Impact of Onchocerciasis Control Through the African Programme for Onchocerciasis Control: an Overview. Annals of Tropical Medicine & Parasitology 92: 8.
- Ubachukwu PO (2006) Socio-Economic Impact of Onchocerciasis with Particular Reference to Females and Children: A review Animal Research International 3: 11.
- Hotez P, Ottesen E, Fenwick A, Molyneux D (2006) The Neglected Tropical Diseases: The Ancient Afflictions of Stigma and Poverty and the Prospects for their Control and Elimination. In: Pollard AJ, Finn A, editors. Hot Topics in Infection and Immunity in Children. New York: Springer. pp. 11.
- World Health Organization (2008) Community Directed Interventions for Major Health Problems in Africa Switzerland.
